# Specificity of a whole blood IGRA in German nursing students

**DOI:** 10.1186/1471-2334-11-245

**Published:** 2011-09-19

**Authors:** Anja Schablon, Roland Diel, Genia Diner, Ute Anske, Wulf Pankow, Felix C Ringshausen, Albert Nienhaus

**Affiliations:** 1 University Medical Center Hamburg-Eppendorf, Institute for Health Service Research in Dermatology and Nursing - Hamburg, Martini Str. 42, 20246 Hamburg, Germany; 2 Hannover Medical School, Department of Respiratory Medicine, 30625 Hannover, Germany; 3 Vivantes Institute for Corporate Health und Safety, Oranienburgerstr. 285, 13437 Berlin, Germany; 4 Vivantes Medical Centre Neukölln, Internal Medicine and Pneumology, Rudower Straße 48, 12351 Berlin, Germany

## Abstract

**Background:**

Interferon-gamma release assays (IGRA) are used for tuberculosis (TB) screening in healthcare workers (HCWs). However, data on specificity of IGRA in serial testing of HCWs is sparse. Therefore the specificity and the negative predictive value of the IGRA - QuantiFERON-TB Gold In-Tube (QFT) - in German nursing students was investigated.

**Methods:**

194 nursing students at the start of their professional career were tested with the QFT. 14 nursing students were excluded from the specificity analysis, due to exposure to mycobacterium tuberculosis. Two of these subjects were QFT- positive. None of them developed disease during the year of follow-up. A study group of 180 students, all with very low risk of prior TB infection, remained in the specificity analysis. Subjects were monitored for at least two years with respect to the development of active TB disease. IGRA was performed at the start of the training and after one year.

**Results:**

The mean age of the study group (n = 180) was 23 years (range 18-53) with 70.9% female and 99.4% German born. The specificity of QFT was 98.9% (178/180; 95% CI 0.96-0.99); lowering the cut-off from 0.35 IU/ml to 0.1 IU/ml would have decreased specificity only slightly to 97.8% (176/180; 95% CI 0.94-0.99). Of the 154 nursing students available for re-testing, one student who initially scored positive reverted to negative, and one student initially negative converted to positive. None of the monitored group with initially negative QFT results developed TB disease, indicating a high negative predictive value of the IGRA in this population.

**Conclusions:**

Following our data, QFT can serve as an effective tool in pre-employment TB screenings for HCWs. As its negative results were stable over time, specificity of the QFT in serial testing of HCWs is high. As the risk of acquiring TB infection in the German healthcare system appears to be low, our data supports the recommendation of performing TB screening only in those HCWs with known contact to TB patients or infectious materials.

## Background

Screening healthcare workers (HCWs) for latent tuberculosis infection (LTBI) and active tuberculosis (TB) disease is a fundamental aspect of infection control programmes in hospitals [1]. The tuberculin skin test (TST) was the first method available for detecting LTBI. However, the TST has known limitations, including cross-reactivity with bacillus Calmette-Guérin (BCG) and non-tuberculous mycobacteria (NTM) infections [2]. Recently, new in-vitro assays have been developed that measure interferon (IFN)-γ released by sensitised T cells after stimulation with *Mycobacterium tuberculosis *antigens. These tests are more specific than the TST since they use antigens not shared by any of the BCG vaccine strains and only by few NTM species (namely M. marinum, M. szulgai, M. kansasii, and *M. riyadhense *[3] that are still rarely detected in clinical settings, but that often have clinical relevance [4] Although in most slow-growing NTM-species ESX-5, a duplication of RD1 and its secretion system (ESX-1) has been detected, the secretion products seem to be secretion system specific [5] and thus do not seem to influence the specificity of the IGRA-assays. Interferon-γ release assays (IGRAs) also have the advantage of correlating better with surrogate measures of exposure to *M. tuberculosis *[6-8] and have a higher predictive value for LTBI progression to active TB disease in close contact in low-incidence settings [9].

Despite a steadily increasing flood of studies on the use of interferon gamma release assays (IGRAs) in detecting latent TB infection (LTBI), the number of studies evaluating specificity, i.e. measuring a test's ability to score LTBI-free subjects as test-negative, is small [10]. Due to the lack of a gold standard to ascertain LTBI in a study individual, specificity can only be measured in healthy subjects who report no known risk for prior *M. tuberculosis *(MTB) exposure and - as far as possible - in an environment that itself offers little or no chance of spontaneous, unrecognised transmission to the test population. Therefore, studies to test a hypothesis of high specificity should be conducted in low prevalence countries where the risk of unnoticed exposure to MTB can be considered negligible. As a more specific test will produce fewer positive results than a less specific test with identical sensitivity, doubts may arise as to the sensitivity of the more specific test. In the absence of a gold standard, reliable proxy measurements for sensitivity are needed. The negative predictive value (NPV), i.e. the probability that a test-negative person is actually negative, is one such proxy for sensitivity. In the case of tests for LTBI, where the consequence of a false negative test may only become apparent long after the test date, the NPV of a test can only be measured in terms of its NPV for progression to active TB disease. As HCWs may be subjected to serial testing for LTBI over the course of their careers, high specificity of the test applied is of particular importance. Some paper gave concern to the specificity of the QFT as they observed high conversion and reversion rates. Therefore we examined the accuracy and specificity of the QFT in a follow-up study on low exposed students entering nursing schools in a low- incidence country.

## Methods

### Study design

Our study group consisted of nursing students (18 years of age or older), trained for work in nursing homes and hospitals, starting their professional career in healthcare institutions operated by the healthcare provider Vivantes (*Institut für berufliche Bildung im Gesundheitswesen (IbBG) Vivantes) in Berlin. Participation was on a voluntary basis. Participants were asked to complete a standardised questionnaire before starting their career in healthcare; questions addressed national origin, whether foreign born, how long they had been living in Germany, possible risk factors for prior exposure to MTB, history of TB disease, inclusion in TB contact tracing exercises in the past, and BCG vaccination status.

One year after enrollment, a second QFT was performed and a new standardised questionnaire was implemented, soliciting information as to specific activities undertaken in the course of the year. Questions addressed the wards or fields of healthcare to which the participants had been assigned, the time period spent there, contact with TB patients and, if any, type and duration of contact. In addition, participants were quizzed on non-professional TB risks such as cases of TB disease in the family or among friends and acquaintances, or journeys of two weeks or longer to high-incidence countries during the first year of work. Each individual was monitored with respect to the development of TB disease for a further year.

Student nurses with risk factors for prior TB infection, including birth in a country with a high TB prevalence, were not taken into account during the analysis of specificity. The study design was approved by the Ethics Committee of the Hamburg Medical Council in Hamburg, Germany.

### Definitions of specificity and negative predictive value (NPV) for progression

Specificity is defined as the number of true negatives divided by the sum of true negatives and false positives. The NPV for progression to active TB is defined as the proportion of test-negative subjects that do not progress to active tuberculosis in a longitudinal follow-up study of individuals tested for LTBI. This value reflects a test's ability to correctly predict that an individual who tested negative for LTBI will not develop active tuberculosis later on in life, assuming that no further exposure to MTB takes place. As only subjects infected with *M. tuberculosis *can develop the disease, the NPV can be measured by following subjects that tested negative for LTBI over time and quantifying the number that have remained free from progression to active disease.

QuantiFERON Gold In-Tube (QFT) was performed according to the manufacturer's instructions (Cellestis Ltd., Chadstone, Australia). As recommended, the cut-off value for a positive test was IFN-γ ≥ 0.35 IU/ml for the TB-antigen minus NIL, provided the NIL control was < 8.0 IU/ml and the TB-antigen minus NIL response was at least 25% of the NIL response. The result was indeterminate if the NIL was > 8.0 IU/ml or if not positive and the mitogen control minus NIL value was ≤ 0.5 IU/ml. All other result profiles provided a negative result. The validated linear range of the QFT ELISA, as reported by the manufacturer, is 0 to 10 IU/ml, thus any value above 10 IU/ml is reported as > 10 IU/ml.

### Statistical analysis

The categorical data was compared using the Pearson's χ^2 ^test or Fisher's exact test, when expected sample sizes comprised fewer than five subjects. The Wilcoxon signed-rank sum test was performed to determine whether the means of two repeated measurements of IFN-γ concentrations differed. All P values reported are based on two-tailed comparisons with statistical significance set at P < 0.05. Statistical analyses were performed using SPSS version 18.0 for Windows (SPSS, Inc., Chicago, IL) and BIAS version 8.3.6 for Windows (Epsilon, Inc., Frankfurt, Germany).

## Results

### Study group at the start

**Figure 1:**
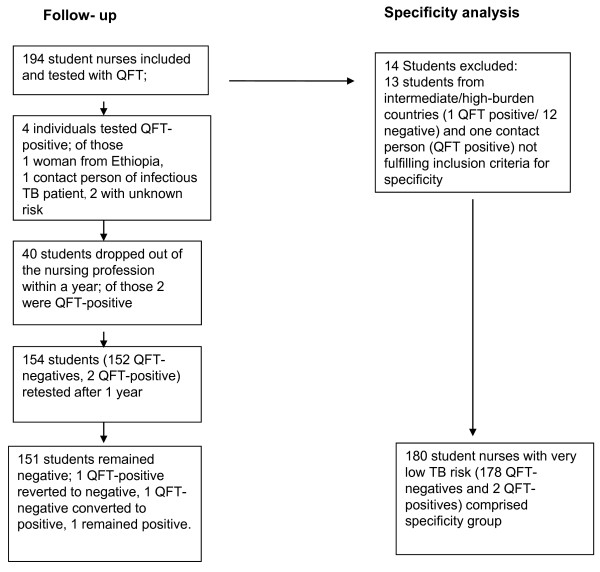
**Steps for building the group for analysing specificity and NPV**.

In all, 194 persons were enrolled between 1 October 2008 and 30 April 2009. The cohort was observed until 30 April 2011 (Figure 1) with a mean period between the first and second IGRA of 17 month (standard deviation 2 month). Of the participants, 104 were distributed to 69 different departments (e.g. gastroenterology, psychiatry, infection ward, etc.) in 33 different hospitals or 68 different departments of these hospitals. The other 90 nursing students worked in numerous nursing homes, rehabilitation centres and mobile nursing services. There were 13 members born in intermediate or high-prevalence TB countries (Cameroon, Poland, Ethiopia, Nepal, Romania, Pakistan, Kosovo, Turkey, Albania, Russia), of whom a 20-year-old woman who moved from Ethiopia to Germany in 2002 was QFT-positive (2.05 IU/ml), all other 12 subjects had negative QFT-results. One 18-year-old German male student had a positive QFT with high INF-γ concentration (> 10 IU/ml) due probably to close contact with an infectious TB patient as this student had worked in healthcare before the training started. However, this link could not be established with certainty. These 14 students were excluded from the specificity analysis. Details of the study population are shown in Table 1.

**Table 1:** Demographic parameters of the 194 study participants

Variables	N	%
Male	58	29.9

Female	136	70.1

Age (years)		

18 to < 25	153	78.9

25 to < 35	26	13.4

35 to < 45	8	4.1

45+	7	3.6

BCG vaccination		

No	93	47.9

Yes	101	52.1

Born in low-incidence country		

No	13	6.7

Yes	181	93.3

German origin		

No	14	7.2

Yes	180	92.8

### Specificity

**Figure 2:**
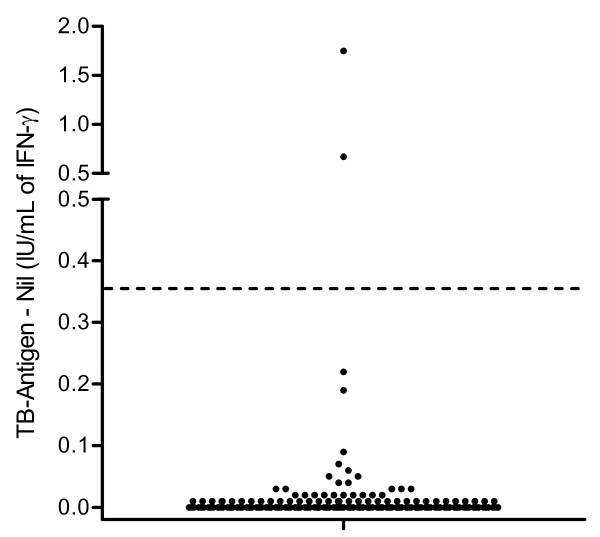
**Dot plot of individual responses to QFT for 180 subjects with valid results and with low risk for TB exposure**. The dashed line represents the cut-off of 0.35 IU/ml for IFN-γ.

Of the remaining 180 subjects with very low risk of MTB infection, two study members, females aged 22 and 42 years, were QFT-positive with a difference of TB antigen-NIL of 1.75 IU/ml and 0.67 IU/ml, respectively. None of the study members had an indeterminate result (Figure 2). None of the study population showed high nil- controls (range from 0.00 to 0.18 IU/ml). Therefore, nobody was IGRA-negative due to the fact of a high nil control. Estimated specificity for the QFT was therefore 98.9% (178/180; 95% CI 0.96-0.999). The mean age of the specificity group was 22.96 ± SD 5.78 (min, max: 18, 53). Members were predominately female (128, 71.1%), and 86 (47.8%) had been BCG vaccinated, of whom BCG vaccination was certificated by a vaccination pass in 85 and one women without certification had a vaccination scar. There was no statistical difference between BCG-vaccines with respect to gender and country of birth. 179 of the low-risk persons were born in Germany, whereas the remaining subject, a 47-year-old man was born in Chile. A lowering of any cut-off value in a test compromises specificity for the supposed benefit of added sensitivity. In our study, if the cut-off (TB AG-NIL) is lowered to 0.10 IU/ml, only two more subjects would be positive with values of 0.19 IU/ml and 0.22 IU/ml; this would reduce our specificity estimate only slightly, to 176/180 (97.8%; 95% CI 0.94-0.99).

### Follow up results

**Figure 3:**
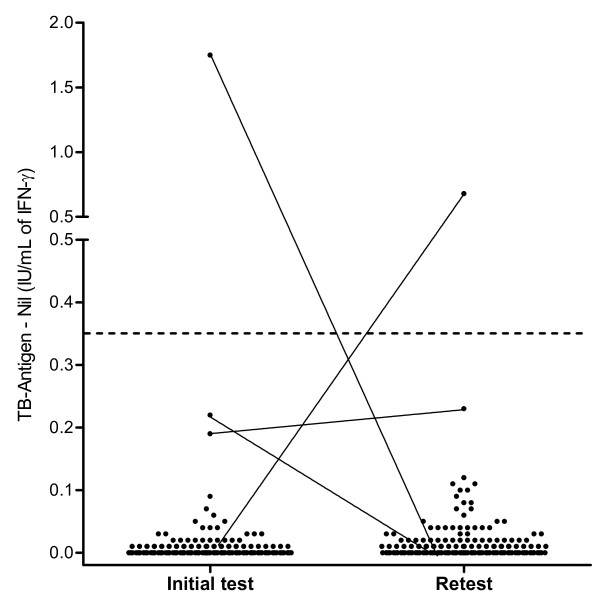
**Dot plot of individual responses to QFT for 154 healthcare workers available for a second test one year after initial testing**. The dashed line represents the cut-off of 0.35 IU/ml for IFN-γ.

Of the 194 study participants initially included in the study, 154 were retested one year after enrollment. The remaining 40 student nurses (20.6%) had dropped out of the training programme. Among the 154 retested healthcare workers, one reverted from a positive to a negative QFT result (from 1.75 IU/ml to 0.00 IU/ml) and one of the persons initially negative converted from negative to positive (from 0.01 IU/ml to 0.68 IU/ml) (Figure 3). The average IFN-γ level of the tested individuals was unchanged between the first testing and the second testing (p = 0.28, n.s.) with a mean of 0.053 ± 0.043 SD in the first and 0.039 ± 0.097 SD in the second QFT. There was no statistical difference between the 40 subjects who dropped out and the remaining 154 HCWs with respect to age, sex, BCG vaccination and origin. 35 of the 154 (22.7%) students had contact to patients with active TB (22 sputum positive patients) during the year between the first and the second QFT tests. However, the student with a conversion in QFT result worked in a nursing home and had no known contact to TB patients (data not shown). Nonetheless, they were followed for progression to active TB for the whole two years. The NPV for progression was 100%, i.e. not a single one of the healthcare workers developed TB disease during the study period

## Discussion

To our knowledge, this is the first specificity study which includes repeated testing and a follow-up period for calculating the NPV of the IGRA. As stipulated by the regulatory frameworks of the respective countries, individuals with contact to infectious TB cases must be investigated so as to prevent the spread of TB disease. However, testing of larger groups of persons in low-incidence countries without any known TB risk is difficult to execute, given ethical considerations. As a result, the number of persons whose presumed lack of infection with MTB is confirmed by a negative IGRA result is relatively low and study groups are quite heterogeneous. The general situation, therefore, does not lend itself to the performance of specificity studies.

Up to now, only six published studies evaluated the specificity of commercially available IGRAs, i.e. QFT and/or T-SPOT.TB, in a total of 736 subjects with valid IGRA results fulfilling the inclusion criteria, i.e. excluding studies performed in intermediate-incidence countries. The study conducted by Detjen et al. included 45 children with confirmed non-TB lymphadenitis or respiratory infections [11], Pallazo et al. [12] included 24 healthy blood donors as controls for TB suspects, and Ruhwald et al. [13] 86 high school students and 38 members of the high school staff. In the paper published by Franken et al. [14] 168 recruits in the Dutch Armed Forces could be considered as having little risk. Wang et al. [15] included 97 adult university volunteers and eleven hospital children, and the study by Bienek and Chang [16] comprised 278 persons in the low-risk group of U.S.-born Navy recruits. According to these studies, the specificity of the IGRAs ranged from 96.9% (95% CI 94.2-98.6%) [10] for the T-SPOT.TB ("European cut-off" without any grey zone) to 100% (95% CI 97.6-100%) for the QFT.

Our study of healthcare trainees just starting their active careers confirms the high specificity of the QFT at nearly 99% and thus presents numbers nearly identical to the estimates of between 98.8% and 100% of the four already published studies in which the specificity of QFT was examined. In addition, however, we retested our individuals after one year and followed them for two years in order to investigate whether a false-negative person not recognised by the test would develop TB disease in the near future. Of the 152 retested persons with initial QFT-negative results who were monitored for at least two years, none fell ill and therefore the NPV for progression was 100%. However, one of the 152 retested participants with an initial QFT-negative result, a student nurse in a nursing home, converted to a positive result. As there was no source of infection identified, the reason for the conversion remains unexplained. Some studies about serial testing in HCWs suggested the use of a grey zone from > 0.2 to 0.7 IU/ml for the QFT results in serial examinations [17-19]. Using this grey zone in our study we found no conversion. As LTBI prevalence in geriatric nurses was reported to be higher than in other HCWs [20] and the student worked in a nursing home, the conversion might be caused by a fresh infection of an unknown source occurring after the first testing. Over the same period, the individual who initially scored positive then reverted to negative.

No further latent TB infection appears to have occurred, although the medical activities of the study participants were carried out in 68 different hospitals (or different departments of those hospitals, including infection wards), in nursing homes and mobile nursing services and 35 students indicated that they had contact to TB patients. This data indicates that in Germany the chance of acquiring TB infection through work in the healthcare sector is low. Schablon et al. [21] tested 2,028 employees in the healthcare sector with the QFT test between December 2005 and May 2009, either in the course of contact tracing or in serial testing of TB high-risk groups pursuant to German OSH legislation, but even then a positive IGRA was found in only 9.9% of the HCWs.

Nevertheless, it was highlighted in a recent systematic review that conversion of IGRA serial testing results is common among HCWs [22]. Two recent serial testing studies among German HCWs found conversion rates of between 1.9% [17] and 5.2% [18]. In Portuguese HCWs the conversion rate was 11% [19]. The higher conversion rate in Portuguese HCWs compared to in German HCWs is in agreement with the higher TB incidence in the Portuguese population. Furthermore, the conversion rates are similar to those described in a meta-analysis for the TST [23]. In the three above-mentioned studies, serial testing was performed because of known contact to TB patients or infectious materials. Only 14.3% of the student nurses had contact to TB patients within the one year between the two QFTs. Exposure was therefore lower than in the two other German serial testing studies [17,18] and a low conversion rate was expected. The conversion rate actually observed was as low as 0.7% (1 out of 153). This indicates a high specificity of the QFT and a good reproducibility of negative QFT results.

The inclusion of a follow-up period in our specificity analysis strengthens our study. However, our study also has limitations. First of all the sample size is rather small. Furthermore, the study was carried out in a population that was not completely free of potential exposure to MTB. But as only one conversion occurred, results were not substantially diluted by performing our study with nursing students.

## Conclusions

Based on our data, it must be asked whether comprehensive serial testing of HCWs following pre-employment screening remains an expedient preventive measure, at least outside of high-risk settings such as TB wards or laboratories. However, as the reproducibility of negative QFT is high, QFT could conceivably be used to monitor the hygiene standards in wards where contact to TB patients or infectious material is to be suspected.

## Competing interests

RD has received reimbursement for attending scientific conferences and/or fees for speaking from Cellestis and Oxford Immunotec. The content of this paper has not been influenced by those companies.

The authors declare that they have no competing interests.

## Authors' contributions

AS participated in the design of the study, was responsible for the data management and was a key party involved in the drafting of. AN conceived the study and has been involved in revising the draft critically for important intellectual content. FCR has been involved in revising the draft critically for important intellectual content. GD and WP participated in the design of the study, made substantial contributions to the acquisition of data and were in involved in writing the draft. UA participated in the design of the study, made substantial contributions to the acquisition of data and was in involved in writing the draft. RD performed the statistical analysis and wrote the draft of the manuscript.

All authors read and approved the final manuscript.

## Pre-publication history

The pre-publication history for this paper can be accessed here:

http://www.biomedcentral.com/1471-2334/11/245/prepub
